# Put a little doxy-PEP in your step: Using doxycycline to prevent chlamydia, syphilis, and gonorrhea infections

**DOI:** 10.1371/journal.ppat.1012575

**Published:** 2024-09-30

**Authors:** Eric A. Meyerowitz, Rina Liang, Derek Bishop, Caroline E. Mullis

**Affiliations:** 1 Department of Medicine, Division of Infectious Diseases, Montefiore Medical Center, Bronx, New York, United States of America; 2 Albert Einstein College of Medicine, Bronx, New York, United States of America; University of Geneva: Universite de Geneve, SWITZERLAND

## Introduction

In 2012, the United States Food and Drug Administration (FDA) approved tenofovir disoproxil fumarate/emtricitabine as pre-exposure prophylaxis (PrEP) to prevent HIV infections in the US, revolutionizing sexual health care and prevention [1]. Comprehensive sexual health clinics have been offering a variety of treatment and prevention strategies since that time (Table 1), though prevention options for bacterial sexually transmitted infections (STIs) like chlamydia, syphilis, and gonorrhea have lagged. On June 6, 2024, the US Centers for Disease Control and Prevention (CDC) recommended doxycycline post-exposure prophylaxis (doxy-PEP) to prevent chlamydia, syphilis, and gonorrhea infections for gay, bisexual, and other men who have sex with men (MSM) and transgender women (TGW) who have had a bacterial STI diagnosed in the past 12 months [2].

**Table 1:** Treatment and prevention strategies for common sexually transmitted infections.

Specific Sexually Transmitted Infection	Prevention Strategy	Treatment
Chlamydia	Doxy-PEP	Doxycycline (antibiotic)
Genital herpes simplex-2 infection	Valacyclovir suppression	Antiviral therapy
Gonorrhea	Doxy-PEP	Ceftriaxone (antibiotic)
Hepatitis A virus infection	HAV vaccine series	Supportive care
Hepatitis B virus infection	HBV vaccine series	Antiviral therapy
Hepatitis C virus infection	None	Antiviral therapy
Human immunodeficiency virus infection	HIV PrEP (TDF/FTC, TAF/FTC, CAB) or HIV PEP	Antiretroviral therapy
Human papilloma virus infection (genital warts, genital cancers)	HPV Vaccine Series	Monitoring, ablation
Bacterial meningitis (*Neisseria meningitidis*)	MenACWY, MenB vaccines (US), MenC vaccine (Europe)	Antibiotics
Mpox infection	Smallpox vaccines	Tecovirimat, supportive care
Syphilis	Doxy-PEP	Penicillin (antibiotic)

CAB, cabotegravir (injectable form of PrEP); FTC, emtricitabine; HAV, hepatitis A virus; HBV, hepatitis B virus; HPV, human papilloma virus; PEP, post-exposure prophylaxis; PrEP, pre-exposure prophylaxis for HIV; TAF, tenofovir alafenamide; TDF, tenofovir disoproxil fumarate.

The efficacy of doxycycline prophylaxis was shown in a US-based multicenter open label randomized trial including 501 MSM and TGW (including 174 living with HIV infection and 327 on PrEP) with at least 1 bacterial STI in the 12 months preceding enrollment [3]. Participants in the doxy-PEP arm were instructed to take a single dose of 200 mg of doxycycline within 72 hours (but ideally around 24 hours) after condomless sex. The primary endpoint was incidence of chlamydia, syphilis, or gonorrhea infections during follow-up, with a median follow-up period of 270 days. The relative risk reductions for chlamydia, gonorrhea, and syphilis infections were 0.12 (95% CI 0.05 to 025), 0.13 (95% CI 0.03 to 0.59), and 0.45 (95% CI 0.32 to 0.65) for people on PrEP and 0.26 (95% CI 0.12 to 0.57), 0.23 (95% CI 0.04 to 1.29), and 0.43 (95% CI 0.26 to 0.71) for people living with HIV infection. Participants were instructed to take no more than 200 mg doxycycline every 24 hours, with 86% of participants reporting consistent use of doxy-PEP after condomless sex and a median number of 4.0 doses per month (IQR 1.0 to 10.0). Two other randomized French studies also demonstrated the efficacy of doxy-PEP at preventing bacterial STIs in MSM and TGW [4,5]. To date, the only study testing doxy-PEP in cisgender women enrolled 449 women in Kenya and found no benefit, but poor adherence may explain these findings as only 29% of participants in the doxycycline arm had evidence of doxycycline in hair samples [6,7]. However, with a median of just 4 doses per month, it is not clear that doxycycline hair levels is the best assessment of appropriate adherence to intermittent and irregular doxycycline dosing [8].

## Logistics

The CDC recommended offering doxy-PEP to all MSM and TGW with a history of chlamydia, syphilis, or gonorrhea in the preceding 12 months. Treatment is 200 mg of any formulation of doxycycline taken one time within 72 hours after condomless sexual encounters. Doxy-PEP may also be considered in MSM and TGW without a diagnosed STI in the preceding 12 months. While pharmacokinetic and STI treatment data suggest doxy-PEP should be effective in other populations, there are no current clinical data showing efficacy of doxy-PEP for cisgender women, cisgender men who have sex with women, or transgender men. The recommendations on doxy-PEP do not extend to populations without efficacy data, but the CDC guidelines acknowledge that clinical judgement and shared decision-making should be used.

Doxy-PEP should be offered as part of comprehensive sexual healthcare. This should include regular screening for chlamydia, syphilis, and gonorrhea, every 3 to 6 months. Chlamydia and gonorrhea screening should occur at any anatomic site used for sex, including the posterior oropharynx, anus, vagina, and penile urethra. It should also include regular HIV screening, or HIV treatment for people living with HIV. HIV PrEP and PEP education and linkage to care services should be provided to all patients without HIV.

Doxycycline is a commonly prescribed antibiotic with many indications besides treatment of STIs, with extremely rare serious side effects. According to a systematic review and meta-analysis, doxycycline is safe for long-term use [9]. The most common side effects are minor dermatologic (i.e., rash) or gastrointestinal (i.e., nausea, vomiting, and abdominal pain) effects. Gastrointestinal side effects can often be mitigated by taking the treatment on a full stomach after a meal. In the large multicenter US doxy-PEP trial, 2% of participants discontinued doxy-PEP due to adverse effects or patient decision [3].

## Modeling studies and early real-world efficacy data

Modeling studies have suggested a profound potential impact of doxy-PEP on incidence of bacterial STIs. An electronic health record analysis including 10,546 individuals with 2 or more bacterial STIs from 2015 to 2020 at an LGBTQ-focused health center in Boston projected a 29% reduction in all bacterial STIs if doxy-PEP were prescribed at the time of the first STI diagnosis [10]. Another Philadelphia-based study projected an 11.6% reduction in syphilis cases with a 20% uptake of doxy-PEP among MSM [11].

The first real-world implementation data are available from San Francisco, which was the first major city to endorse doxy-PEP for MSM and TGW in local guidelines. After implementation of a doxy-PEP program from November 2022 to September 2023, chlamydia and early syphilis cases decreased 51% (95% CI 39% to 60%) and 50% (95% CI 38% to 59%), respectively, among MSM/TGW compared to expected counts for November 2023 [12]. On the other hand, chlamydia cases increased during the follow-up period (2.43% per month) among cisgender women, a population where doxy-PEP was not implemented.

## Looking ahead—Areas of uncertainty

The impact of doxy-PEP on individual and population-level bacterial resistance is an area of active study. In the US-based DoxyPEP randomized trial, baseline tetracycline resistance performed on gonorrhea cultures was 27% (4/15 isolates tested) [3]. After enrollment, 38% (5/13 isolates) in the doxy-PEP group and 12% (2/16 isolates) in the standard-care group were tetracycline resistant. In the DOXYVAC study, there was significantly increased tetracycline resistance in gonorrhea isolates in doxy-PEP users compared to nonusers [5]. In both DoxyPEP and DOXYVAC, there was a smaller absolute benefit for prevention of gonorrhea versus chlamydia and syphilis infections [3,5]. With potential selection and proliferation of tetracycline-resistant isolates, it is possible the benefit of doxy-PEP on prevention of gonorrhea infections seen in these early studies may be extinguished over time. Doxycycline and tetracycline are not recommended for treatment of gonorrhea infections due to presumed baseline resistance.

The impact of doxy-PEP on *Staphylococcus aureus* (*S*. *aureus*) is another area of uncertainty. Oronasopharyngeal carriage was found in 45% of participants at baseline, with 12% of participants carrying doxycycline-resistant strains. Twelve months after doxy-PEP enrollment, overall *S*. *aureus* carriage was lower in the doxy-PEP group (28%) relative to the standard-care group (47%). While overall population-level doxycycline-resistant *S*. *aureus* did not significantly differ (5% in doxy-PEP versus 4% in standard-care group), a higher proportion of those with *S*. *aureus* carriage in the doxy-PEP group had doxycycline resistance (16% versus 8%). The degree to which an increased uptake of doxy-PEP will impact the proliferation of doxycycline-resistant *S*. *aureus* at a population level is not yet known. Fortunately, there are many alternative antibiotics (including oral regimens) that are active against *S*. *aureus*, including doxycycline-resistant strains. The impact of doxy-PEP on emergent resistance in gram-negative organisms is also uncertain. The DOXYVAC study found no difference in extended-spectrum beta-lactamase producing *E*. *coli* in rectal swabs among doxy-PEP users and nonusers [5].

Doxycycline-resistant *Chlamydia trachomatis* infections remain incredibly rare. There have been a few case reports of treatment failure attributed to drug resistance including doxycycline resistance [13]. Close monitoring of chlamydia infections in patients on doxy-PEP is critical to determine whether development of doxycycline resistance becomes clinically relevant. While test-of-cure is not typically recommended for chlamydia infections, it may be reasonable for patients who have a “breakthrough” chlamydia infection while using doxy-PEP.

Doxycycline is an alternative treatment option to penicillin for syphilis infections, with extensive clinical data supporting its efficacy [14]. There is no clinically significant penicillin or doxycycline resistance in *Treponema pallidum* (the bacterial etiology of syphilis infections). In contrast, there has been a proliferation of macrolide-resistant *T*. *pallidum* strains, with >99.2% of North American strains now resistant to this class of antibiotics (up from 53% in 2007) [15]. Close monitoring is needed to monitor for the emergence of doxycycline-resistant *T*. *pallidum* in the doxy-PEP era. Because syphilis testing relies on serologic testing (rather than DNA or RNA testing), doxy-PEP may change serologic performance. For instance, delayed or incomplete seroconversion and seroreversion may be seen in patients taking doxy-PEP [16]. These phenomena have been previously reported, particularly in the setting of successful treatment of very early syphilis infections. The clinical significance of delayed or incomplete seroconversion in the setting of doxy-PEP use is currently unknown, though providers should be aware of this and refer to syphilis treatment experts if unusual results are encountered.

## Conclusions

Like HIV PrEP, doxy-PEP, a 200 mg of any formulation of doxycycline taken one time within 72 hours after condomless sexual encounters, is a transformational prevention strategy endorsed by the US CDC in June 2024 that is being widely implemented in sexual health clinics. Early data suggest that it will have a major impact on curtailing the incidence of bacterial STIs. The degree to which increasing doxy-PEP uptake impacts antimicrobial resistance among bacterial STIs and other unrelated bacteria (i.e., *S*. *aureus*) remains unknown and requires ongoing long-term study. There is an urgent need for rapid STI diagnostics to detect genotypic markers for antimicrobial resistance and for better syphilis diagnostics given the problems and inadequacies of current serologic testing strategies. Additionally, HIV PrEP has been plagued by inequity, with significant racial, gender, geographic, and socioeconomic disparities [17]. Gender equity requires investment in clinical trials of doxy-PEP in cisgender women. Going forward, implementation strategies for doxy-PEP should be rooted in equity and designed to simultaneously increase access to comprehensive sexual health services, including PrEP, and uptake in areas where these services remain scarce and underutilized.

## References

[1] Baeten JM, Haberer JE, Liu AY, Sista N. Preexposure prophylaxis for HIV prevention: Where have we been and where are we going? J Acquir Immune Defic Syndr. 2013 Jul 1;63(Supplement 2):S122–S129. 23764623 10.1097/QAI.0b013e3182986f69 PMC3710117

[2] Bachmann LH, Barbee LA, Chan P, Reno H, Workowski KA, Hoover K, et al. CDC clinical guidelines on the use of doxycycline postexposure prophylaxis for bacterial sexually transmitted infection prevention, United States, 2024. MMWR Recomm Rep. 2024 Jun 6;73(2):1–8. doi: 10.15585/mmwr.rr7302a1 38833414 PMC11166373

[3] Luetkemeyer AF, Donnell D, Dombrowski JC, Cohen S, Grabow C, Brown CE, et al. Postexposure doxycycline to prevent bacterial sexually transmitted infections. N Engl J Med. 2023 Apr 6;388(14):1296–1306. doi: 10.1056/NEJMoa2211934 37018493 PMC10140182

[4] Molina JM, Charreau I, Chidiac C, Pialoux G, Cua E, Delaugerre C, et al. Post-exposure prophylaxis with doxycycline to prevent sexually transmitted infections in men who have sex with men: an open-label randomised substudy of the ANRS IPERGAY trial. Lancet Infect Dis. 2018 Mar;18(3):308–317. doi: 10.1016/S1473-3099(17)30725-9 29229440

[5] Molina JM, Bercot B, Assoumou L, Rubenstein E, Algarte-Genin M, Pialoux G, et al. Doxycycline prophylaxis and meningococcal group B vaccine to prevent bacterial sexually transmitted infections in France (ANRS 174 DOXYVAC): A multicentre, open-label, randomised trial with a 2 × 2 factorial design. Lancet Infect Dis. 2024 May;S1473309924002366. 10.1016/S1473-3099(24)00236-6 38797183

[6] Stewart J, Oware K, Donnell D, Violette LR, Odoyo J, Soge OO, et al. Doxycycline prophylaxis to prevent sexually transmitted infections in women. N Engl J Med. 2023 Dec 21;389(25):2331–2340. doi: 10.1056/NEJMoa2304007 38118022 PMC10805625

[7] Stewart J, Donnell D, Violette LR. Self-reported adherence to event-driven doxycycline postexposure prophylaxis for sexually transmitted infection prevention among cisgender women. STI and HIV World Congress. 2023 Jul 24.

[8] Doxycycline to prevent sexually transmitted infections in women. N Engl J Med. 2024 Apr 4;390(13):1248–1249. 10.1056/NEJMc2401273 38598593

[9] Chan PA, Le Brazidec DL, Becasen JS, Martin H, Kapadia J, Reno H, et al. Safety of longer-term doxycycline use: A systematic review and meta-analysis with implications for bacterial sexually transmitted infection chemoprophylaxis. Sexual Trans Dis. 2023 Nov;50(11):701–712. doi: 10.1097/OLQ.0000000000001865 37732844 PMC10592014

[10] Traeger MW, Mayer KH, Krakower DS, Gitin S, Jenness SM, Marcus JL. Potential impact of doxycycline post-exposure prophylaxis prescribing strategies on incidence of bacterial sexually transmitted infections. Clin Infect Dis. 2023 Aug 18;ciad488. doi: 10.1093/cid/ciad488 37595139 PMC13016692

[11] Tran NK, Goldstein ND, Welles SL. Countering the rise of syphilis: A role for doxycycline post-exposure prophylaxis? Int J STD AIDS. 2022 Jan;33(1):18–30. doi: 10.1177/09564624211042444 34565255 PMC8688295

[12] Sankaran M, Glidden D, Kohn R, Liebi C, Torres T, Buchinder S, et al. Doxy-PEP associated with declines in chlamydia and syphilis in MSM and trans women in San Francisco [Abstract 127]. CROI 2024 [Internet]. [cited 2024 Jul 10]. Available from: https://www.croiconference.org/abstract/doxy-pep-associated-with-declines-in-chlamydia-and-syphilis-in-msm-and-trans-women-in-san-francisco/

[13] Somani J, Bhullar VB, Workowski KA, Farshy CE, Black CM. Multiple drug–resistant *Chlamydia trachomatis* associated with clinical treatment failure. J Infect Dis. 2000 Apr;181(4):1421–1427. 10762573 10.1086/315372

[14] Workowski KA, Bachmann LH, Chan PA, Johnston CM, Muzny CA, Park I, et al. Sexually transmitted infections treatment guidelines, 2021. MMWR Recomm Rep. 2021 Jul 23;70(4):1–187. doi: 10.15585/mmwr.rr7004a1 34292926 PMC8344968

[15] Lieberman NAP, Reid TB, Cannon CA, Nunley BE, Berzkalns A, Cohen SE, et al. Near-universal tesistance to macrolides of *Treponema pallidum* in North America. N Engl J Med. 2024 Jun 13;390(22):2127–2128. 38865666 10.1056/NEJMc2314441 PMC11345850

[16] Raccagni AR, Bruzzesi E, Castagna A, Nozza S. Doxycycline postexposure prophylaxis may delay seroconversion in incident syphilis. Sex Transm Infect. 2024 Jun 17;sextrans-2024-056240. doi: 10.1136/sextrans-2024-056240 38886053

[17] Sullivan PS, DuBose SN, Castel AD, Hoover KW, Juhasz M, Guest JL, et al. Equity of PrEP uptake by race, ethnicity, sex and region in the United States in the first decade of PrEP: A population-based analysis. Lancet Reg Health Am. 2024 May;33:100738. doi: 10.1016/j.lana.2024.100738 38659491 PMC11041841

